# Affectivity and Sexuality in Adolescents with Autism Spectrum Disorder from the Perspective of Education and Healthcare Professionals: A Qualitative Study

**DOI:** 10.3390/ijerph20032497

**Published:** 2023-01-31

**Authors:** Jordi Torralbas-Ortega, Victoria Valls-Ibáñez, Judith Roca, Meritxell Sastre-Rus, Carme Campoy-Guerrero, Dolores Sala-Corbinos, María Sánchez-Fernández

**Affiliations:** 1 Hospital de la Santa Creu i Sant Pau, Nursing Care Research Group, Sant Pau Biomedical Research Institute (IIB SANT PAU), 08041 Barcelona, Spain; 2 Health Center La Serra, Catalan Health Institute, 08208 Sabadell, Spain; 3 Department of Nursing and Physiotherapy, University of Lleida, 25198 Lleida, Spain; 4 Research Group of Health Care (GRECS), IRB Lleida, 25198 Lleida, Spain; 5 Child and Adolescent Day Hospital, Sant Joan de Déu-Terres de Lleida, 25001 Lleida, Spain

**Keywords:** autism, autism spectrum disorder, sexuality, affectivity, healthcare professionals, education professionals

## Abstract

People with autism spectrum disorder (ASD) present several different characteristics that predispose them to greater difficulties with intimate emotional and sexual relationships. This qualitative study uncovers the perspective of education and healthcare professionals on the affective and sexual needs of young people with ASD by analysing their narratives in semi-structured focus group sessions. Professionals highlight the inadequate training they receive in this aspect of health. They consider it should be commonplace for professionals working with autistic people as it would aid their collaborative efforts when treating children and adolescents with ASD. They show that, by working together with the families to establish joint objectives, these professionals can appropriately address sex and affective education, preventing risky behaviours among young people with ASD, and improving the interactions these individuals have with others. Sex and affective education is described as an indispensable tool at this stage of development and should be specially adapted for those with ASD.

## 1. Introduction

Autism spectrum disorder (ASD) affects 1.23% of the child and adolescent population in Catalonia, more so among males than females, with a ratio of 4.5:1, respectively [1]. ASD is a neurodevelopmental disorder, as defined in the Diagnostic and Statistical Manual of Mental Disorders, or DSM-5 [2], which manifests in a wide variety of ways, both between individuals and in a single person throughout their development. Initially, it manifests as impairments in the development of reciprocal social interaction, verbal and non-verbal communication, and a limited repertoire of interests and behaviours [3].

Although early diagnosis and treatment can improve cognitive, social, and communicative function, symptoms and difficulties generally persist throughout adolescence and into adulthood [4]. One of the main focuses of work in this field is both formal and informal socialisation to encourage healthy behaviours in all aspects of life. Socio-affective learning is highly complex and, as such, is easier to address in formal situations such as academic settings, where it can thus be more fruitful [5,6,7,8].

Within the sphere of affective development, sexuality has been largely ignored; perhaps due to the fact that young people with ASD are often infantilised and considered asexual, or lacking interest in intimacy [5]. However, studies suggest that adolescents and young adults with ASD desire and search for affective and sexual relationships, taking part in a variety of sexual behaviours typical of most young people [9,10,11].

Affective and sexual relationships can be a valuable focal point in order to improve the quality of life of individuals with ASD and the people around them, primarily because they have an increased risk of negative consequences, such as sexual abuse, loneliness, unwanted pregnancy, sexually transmitted diseases, and inappropriate sexual behaviour [12,13,14].

Changes in contemporary sex education (e.g., more democratic educational styles, greater freedom for sexual expression, easier access to sex information, and so on) have facilitated the adaptation of these processes in current thought, but there are still some challenges regarding the assimilation of individuals with ASD due to the very nature of their condition, such as rigid thinking and the lack of flexibility regarding established norms [3,12]. Furthermore, some authors highlight that autistic adolescents and adults have reduced sexual knowledge and awareness [15]. As such, professionals who regularly work with these individuals can play a crucial role in developing a healthy sexuality, while minimising and monitoring risk thanks to providing education, tools, and decision-making support systems for young people and their families, in order to overcome the challenges of adolescent and young adult life [16]. The family is an invaluable resource to establish boundaries and provide sex education to individuals with ASD. However, they should be aware of the specific needs of their child and the risks that they face, receiving the support they need from education and healthcare professionals [17,18]. Consequently, it is pertinent that we explore the experiences of education and healthcare professionals with regards to sex and affective education of young people with ASD.

## 2. Materials and Methods

### 2.1. Study Design

This study used a descriptive, qualitative design [19]. The aim of qualitative description is to collect the maximum amount of data possible for a comprehensive look at the object of study, even though said description does not exclude data interpretation on the researcher’s part [20]. By describing the experiences and perceptions of participants—being education and healthcare professionals in this study—we can encourage the improvement of aspects of education and professional practice.

### 2.2. Setting and Particpants

The study was carried out at the Consorci Corporació Sanitària Parc Taulí [Parc Taulí Hospital] in Sabadell, Barcelona. It is a tertiary healthcare facility that is part of the Catalan Health Institute care network, currently attending to a population of 493,000 citizens, and is thus under public management. The Mental Health Area “https://www.tauli.cat/salutmental/usuaris/informacio-i-consells-als-usuaris (accessed on 26 January 2023) provides universal coverage and access to healthcare for children, adolescents, and adults with various psychological disorders. Patients are recruited through outpatient consultations, emergency admissions, or referrals from other specialties. Users are in direct contact with mental health professionals and education professionals working with children and adolescents in special and non-special schools. Interaction between these professionals takes place through interdepartmental coordination in the area of reference at each mental health facility.

Education and healthcare professionals who work closely with individuals with ASD were invited to take part in focus group sessions using purposive sampling. Professionals with long-term work experience (more than 5 years) in the field were included in order to guarantee a minimum degree of prior exposure to the phenomenon being studied. With the intention of maximising the diversity of perspectives in the sample, individuals from various disciplines and roles were included (in management and healthcare, for example). A total of 18 participants were included in four different focus groups, which were analysed at the end of each session individually until data saturation was reached. The participant descriptions are shown in Table 1.

### 2.3. Data Collection

The data collection technique used was focus groups. The thematic script to guide focus group sessions included questions that introduced the study dimensions, which were defined based on the literature review findings and the experience of the research team. The following dimensions were introduced to the group in the questions: family; training; sex education; and the perceived sexuality of individuals with ASD. Table 2 shows the script for the focus group sessions.

After contacting the participants directly, an appointment was set (place and time) for each focus group. These groups were led by expert professionals who were independent of the investigation study. The participants were thanked for taking part, informed that the discussion would be recorded, and each person had to sign an informed consent form to continue participation in the study. The focus group sessions were performed between March and May of 2019, lasting between 60 and 90 min, and were recorded from start to finish. The observations and incidences that occurred during the execution of these sessions were recorded in a field notebook and inserted into the transcriptions.

The fact that the majority of the research team was part of the study group could have changed the way the interviews were carried out. To avoid this, four experts were introduced into the research team, who were trained in carrying out this particular technique but were not related to the context of the study. They were responsible for leading the four focus group sessions: two for education professionals and two for healthcare professionals.

### 2.4. Information Analysis

The content analysis of the collected information was carried out by the research team, following the guidelines suggested by Graneheim et al. [21,22] using the NVivo v12 program.

First, the information from all four focus groups was transcribed literally. Subsequent reading and re-reading allowed for identification of the units of analysis. Next, a coding structure was established in which the codes, categories, and themes were created from recurring elements and specific objectives. This structure was applied systematically to all of the transcriptions.

Quality and rigour (credibility, dependability, and transferability) were ensured through triangulation strategies, saturation, and validation by informants. The transcriptions were analysed independently by two researchers, and then in a group session, where the thematic areas from each analysis were discussed and agreed upon.

### 2.5. Ethical Considerations

This study was assessed and approved by the Clinical Research Ethics Committee at the Parc Taulí Hospital, with reference number 2018509. The anonymity of the informants was respected at all times. Each participant was assigned an alphanumeric code. All names that may have identified education and healthcare professionals and/or their respective places of work were removed from the text.

## 3. Results

### 3.1. Experiences of Education Professionals

Table 3 details the topics and categories that emerged from the narratives of the focus group sessions carried out with education professionals.

#### 3.1.1. Family Coping

Family is one of the key pillars in adolescent development and socialisation, also providing social and cultural knowledge. The young person and their family work together as a team and it is necessary to identify the factors that can help their sex and affective education.

Family beliefs about having ASD means they think that their children will not have any affective and sexual needs and, as such, they do not consider it necessary to address nor educate their children about these needs.

*E5 ‘And that’s the difficulty a lot of families have, and they keep putting it off, and by the time we in schools are already dealing with the problem left, right and centre... it’s already a need and they have a lot of pent-up, nervous energy, they have to learn about it. So, you just have to work on it with the family. Many times, we make it something official from the school because they really struggle to wrap their head around the issue.’*

In relation to family difficulties, intrinsic familial factors such as culture, level of education, and prejudices about sexuality make it difficult for them to guide their children towards the skills they need to develop a healthy sexuality. In addition, overprotectiveness and the lack of knowledge and skills generate resistance to these discussions, delaying having to address this topic.

*E3 ‘Because you can give them resources, but emotionally, if they’re not able to support their child in this process, they don’t [...]. Parents surprise themselves, they really struggle, like, we’ll cross that bridge when we come to it...’*

*E5 ‘The most interesting thing about the families of students at special schools is that they’re overprotective. Their kids are kept in a bubble, and when that inevitable push comes during adolescence or preadolescence, the family can’t accept it at first.’*

Both family denial and the fantasy of having normotypical relationships, especially in early childhood, act as hurdles when attempting to address topics relating to affectivity and sexuality of young people with ASD and their potential vulnerability.

*E1 ‘sexuality doesn’t exist... when the first problems or maladaptive behaviours come up, or things that haven’t been addressed, the alarm bells start ringing.’*

*E5 ‘the “de-education” in this sense comes directly from the families. This is one of the most concerning factors because there are people, there are young people who can be easily victimised, which makes them vulnerable.’*

In some cases, families even react to their fear by passing them off onto their children, which is an extremely maladaptive and limiting response.

*E1 ‘then what they do is make them afraid, put limits on them, start forbidding things...’*

The presence of siblings can be seen as a positive influence within the family unit, as it is a way of starting the discussion about sexuality.

*E3 ‘for them it’s awkward, so it helps having a sibling in the family, but if the mother or father has to step in it becomes even more awkward, and if the parent doesn’t understand the dynamics, then it can be difficult to handle.’*

#### 3.1.2. Training for Education Professionals

Analysis of the specific training category addresses the perception of education professionals towards their training and needs. Educators report the training they received as being not very specialised, not taking the details of this topic into consideration, and the need to train themselves through experience.

*E5 ‘The training was quite general; it didn’t cover any particular profile of students with ASD.’*

*E1 ‘I don’t have any training on this topic and I’ve never been offered training on sexuality among students with ASD.’*

Furthermore, training needs are based on offering sex and affective education within the curriculum with only very basic training, addressing different behaviour patterns, and obtaining access to professional resources that can help both the adolescents in question and their families.

*E4 ‘to be able to do certain things that should be soft skills within a primary or secondary curriculum, adapted or not, because it’s a part of being human, but it’s quite a... neglected topic.’*

*E1 ‘specific sexual behavioural patterns, how to handle them, and obviously, how to interpret both our role and the family’s role in this whole process.’*

#### 3.1.3. Curricular and Collaborative Strategies

Current processes to address affectivity and sexuality in educational learning centres are not consistent across special education nor non-special education. Teaching staff affirm that they need suggestions so that there is a cross-sectional approach to the discussion and so that it becomes another area within the educational curriculum for young people, and in which the families can play an active role in the process.

*E1 ‘our school’s curriculum involves the families in affectivity and sexuality education. We periodically find resources to give talks on these topics.’*

Coordination with families is vital but complex, as families prefer individual treatment over work in group sessions, making attendance at group sessions low.

*E5 ‘with families, we also have quite low participation, and we really struggle to promote talks, whether about sex-affectivity or any other topic, so we also have to take advantage of our trimestral tutorial sessions.’*

Finally, teachers recognise the need for multidisciplinary collaboration with healthcare staff for the benefit of both students and their families.

*E3 ‘when we’ve had a dedicated school nurse with previous experience, it’s much more effective than what we could have done as teachers [...], so that’s why we also need support from medical professionals to guide families.’*

#### 3.1.4. Profile of Adolescents with ASD with Regards to Their Affective/Sexual Development

Teachers also identify categories relating to inappropriate social behaviour; these include non-normative behaviour and difficulty interpreting various social situations.

*E2 ‘in a playground I came across a boy, who started to masturbate as he approached me.’*

*E1 ‘for example, complex social situations with classmates, uncontrollable verbal outbursts... they don’t know where the limits are.’*

*E1 ‘the relationships that one boy with high-functioning ASD had with his partners repeatedly followed a pattern of excessive possessiveness.’*

Another aspect to keep in mind is risky behaviours, especially among girls with ASD, which puts them in more vulnerable situations.

*E2 ‘Four days after the sweet falling in love stage the possessiveness comes out, you know? The role of the dominated woman that almost all of the female students played.’*

There are also communication problems. Communication becomes much more complex; it is important to be able to understand other codes or expressions. At times, it can be difficult to understand the message being transmitted.

*E4 ‘Children with ASD don’t communicate with one another, and if they do, they don’t do it explicitly... they use another code... on a different wavelength. It’s not your everyday communication.’*

*E2 ‘they have less autonomy, express mental needs in a more physical way, with more gestures, fidgeting [...], even self-stimulation.’*

Adolescents with ASD experience a problematic and dysfunctional sexuality, as they are unable to identify feelings, find decision-making difficult, have a lack of skills, and are unsatisfied with sexual practices.

*E2 ‘Physically they know that things are happening to them, but they don’t know what, what to do in response, or how. They also don’t know how to put a name to the feelings and sensations they are having.’*

*E5 ‘very vulnerable situations compared to others, which are maybe hypersexualised, you know? They [also] have difficulties with decision-making, they can’t choose between one or the other [...], or they feel the need to masturbate but they don’t know when to stop.’*

Furthermore, knowledge acquisition through appropriate channels allows these young people to have more realistic expectations. Adolescents who decide to look for information often do so through the internet and, as such, the information they find is often distorted and far from healthy sexual behaviours.

*E4 ‘teenagers with easy, unlimited access to the internet, who discover what they find pleasurable or not, and from that, they start building a sexuality that can be harmful.’*

*E3 ‘when you have closer contact, you sometimes see an excessive consumption of pornography: I’m talking about a student with high-functioning ASD with very sadistic sexual practices a lot of the time.’*

However, this resource can also be an excellent tool to increase knowledge when used under professional or family supervision.

*E5 ‘we also work a lot with explicit visual supports: who can touch me, which parts other people can touch, which they can’t, all depending on the type of relationship.’*

*E3 ‘role-plays really worked for us, social scenarios to help anticipate different situations.’*

### 3.2. Perception of Healthcare Professionals

The themes and categories extracted from the healthcare professionals focus groups are shown in Table 4.

#### 3.2.1. Experiences with the Family

It should be noted that interactions between healthcare professionals and families are shorter and less frequent compared with those between education professionals and families. Healthcare professionals highlight that consultations of this nature are not common, and reasons for consultation are often either inappropriate behaviour of adolescents when they have a social, family, or relationship issue or the lack of knowledge among families. In addition, families often handle their lack of knowledge by turning to a professional to approach the topic with their child.

*H5 ‘I don’t know much and I don’t want to talk about it, or I’m too scared, or I don’t know. There’s a gap in my knowledge. And... and so I’m bringing them to you.’*

*H8 ‘It can also happen that we’re all there sitting down and they start to masturbate because they say they feel comfortable.’*

Healthcare professionals also suggest that cultural diversity is a variable that influences how affectivity and sexuality are addressed and experienced. This cultural diversity reinforces the connection to other factors, such as level of knowledge, experience with sexuality, or even possible abuse.

*H9 ‘various cultural groups are represented here. The care I provide to a young, Romani girl who is going to get married and doesn’t know what a sexual relationship is... [compared to the care I could give] a girl from Morocco, for example, or a local girl.’*

*H5 ‘If you talk to an African girl, sexuality doesn’t exist. Sexual affectivity doesn’t exist, it’s just a way to procreate.’*

*H5 ‘Within that same marriage there is abuse that is tolerated for cultural reasons.’*

When discussing the cultural diversity of our area during the focus group sessions, participants refer to people’s general ethnic/cultural background as a way of exemplifying cultural differences in sexuality without going into specific detail about their examples. It goes without saying that these are broad generalisations that do not apply to every person belonging to that particular ethnic/cultural collective. However, we believe that cultural facts and differences regarding sexuality, such as taboos and boundaries, are relevant to sex and affective education. They have specific ways of being dealt with in different cultures, countries, and regions around the world, thus meriting the inclusion of the participant examples. Moreover, cultural differences are a frequent factor to be considered among healthcare professionals when carrying out their daily work.

#### 3.2.2. Specialised Training of Healthcare Professionals

Professionals mention that the training they receive is broad and insufficient, and there is a lack of studies and research that can actually inform professional practice.

*H9 ‘because we can assume things, but in reality, we don’t have a lot of real information or literature on this topic, you know?... Neither quality research nor research on this topic.’*

*H8 ‘to be able to speak with authority and for studies to discuss sexual conduct of individuals with ASD... I mean, there has to be some kind of model to guide professionals in this field, like there are for other situations.’*

This lack of information means that exploration of sexual/affective topics during consultations is not enough. However, professionals are aware of the problem and the need to broach this topic from infancy with adolescents and their families, just like any other therapeutic situation.

*H8 ‘Sometimes we ask if they have a partner or not, but we don’t ask if they’re sexually active, even though it’s useful data.’*

*H3 ‘We work with groups of parents on how they can approach the topic and work on it with their children.’*

#### 3.2.3. Profile of Adolescents with ASD

Healthcare professionals note that there is a lack of knowledge among young people with ASD regarding affective and sexual relationships, and the acceptance of changes they experience during puberty.

*H8 ‘they also don’t know much about, nor accept, things like growing hair, for example in boys... The question is, how do we do this right?’*

In addition, adolescents with ASD do not interpret relationships in the same way as neurotypical adolescents. This can lead to conflict situations with other teenagers.

*H10 ‘someone says “I like your jumper” and they take that to mean they’re together now.’*

As such, these adolescents have the capacity to be abused or be the abuser. Girls are more likely to be mistreated and boys exert a dominant role and, as such, it is important to educate them based on these specificities while keeping in mind the huge degree of vulnerability that these groups face.

*H2 ‘[It’s] especially [necessary] to empower them to say no, [as] that’s closely related to mistreatment, abuse...’*

*H3 ‘Many times they know it’s not right [their behaviour] but they also don’t have the skills or the confidence to explain it, and they end up with bad experiences.’*

*H1 ‘You can show them how to prevent what could be invasions of privacy or sexual aggression, you know? But you’re always left thinking what would happen outside of a session? In a group at the community centre? What if one day in a shop somewhere...? You just don’t know.’*

## 4. Discussion

In this study, we investigated the perceptions of education and healthcare professionals in regards to adolescents with ASD. The findings show that both parties agree that it is necessary to work together with families to be able to discuss sex and affective education of their children from infancy. On the one hand, it would facilitate the recognition of affective and sexual needs of this collective, and educational content could be adapted to the biological maturation of each individual and their specific needs. On the other hand, identifying these specific needs would allow us to anticipate more complex situations. As other experts have shown [23,24,25], providing tools to parents and young people as well as key figures (such as siblings) for managing this need will allow individuals with ASD to have healthier affective and sexual relationships. However, there are few studies that mention specific programmes that broach this topic [26]. In the family context, in order to address the needs or difficulties identified (overprotection, denial, fear, and so on), other authors [27] suggest identifying positive areas of adaptation such as facilitating beliefs, empowering support networks, and family engagement. Sexuality is a sensitive topic for families and involves discussions that will vary according to cultural aspects, family values, and beliefs [18].

Both professional groups agree that there is a need for specialised training on affectivity and sexuality from experts in the field, as it would allow them to better approach this topic both with parents and students or other users. Early-education programmes ought to be created in this field that could later lead to a cross-sectional, comprehensive, depathologising approach in education and in healthcare [28,29]. Teachers should facilitate the educational process under the paradigm of curriculum accessibility rather than curriculum adaptation [30], which requires elements of support and integration. Interventions must be designed to integrate students with disabilities and special needs such as ASD into academic and social activities in an open and programmed way, which enhances their resilience and learning skills, thus breaking away from the so-called ‘hidden curriculum’ [31]. In addition, research suggests that promoting social communication skills results in improved language use and communication skills in general [32].

Teachers highly value coordination and collaboration with healthcare professionals. According to some researchers [18], teachers propose a programme in which both groups would collaborate on an educational level to broach specific topics with students and their families. In addition, the interaction between adolescents with ASD and educators is more intense and, as such, concrete support is needed to provide the appropriate tools for opening discussion [33]. However, healthcare professionals are more aware of the need to approach this question on an individual scale with each patient and their family in various healthcare consultations, as well as from a health promotion standpoint in group therapies, to minimise problems and conflicts in adolescence and adulthood [25]. In the narratives of professionals, the impact of culture also emerges as an important aspect to consider and investigate in contexts where sexuality is mediated by families [18].

Adolescents with ASD have specific traits that are inherent of their neurodiversity, making them perceive the world in a different way, and making them vulnerable to abuse—both as the abused and the abuser. Learning about these traits would provide us with indicators to monitor both the risks and needs of each individual [34]. Knowledge is a protective factor that allows young people to learn to have healthy affective and sexual relationships, and identify what they do or do not want for both themselves and others. The improvement of interpersonal and social skills can have an impact on communication and empathetic deficits [35]. It should also be highlighted that studies such as Sala et al. [9] show that individuals with ASD are able to reflect on their own sexuality.

With the gender perspective focusing on the under-recognition of ASD in women, it has also highlighted the profile of adolescent girls with ASD, with professionals perceiving them as more vulnerable. This profile, characteristic of an easily dominated person, requires a more intensive approach to education and skill acquisition, as reported by families and professionals [36]. Conversely, males with dominant characteristics need more support in establishing relationships founded on equality and respect.

The current sex education that young people receive, focused on the immediacy of social media and the increasing distortion of reality, should make us think more about what kind of sex and affective education we want for our society. This is especially true for people who are more vulnerable in social relationship situations [37,38]. Finally, we should highlight that sexuality is relevant for health development and quality of life for adolescents with ASD, and it requires additional education to achieve optimal development of affective and sexual aspects of their growth [39].

## 5. Conclusions

There is a need for targeted education of adolescents with ASD with regards to affective and sexual matters from infancy, as well as for their families. The content of this education should be adapted to the particularities of ASD and should include multidisciplinary collaboration between education and healthcare professionals.

Vulnerability in the affective and sexual sphere of young people with ASD, as it is perceived by these professionals, should be a fundamental element in establishing an educational curriculum for sexual health in each of these young people’s environments, thereby allowing for a comprehensive and cross-sectional approach to their education.

To achieve this, specialised training is needed for all professionals who integrate education into the healthcare of these young people, as well as better internal coordination of the needs of these adolescents in distinct areas.

## Figures and Tables

**Table 1:** Focus group participants.

Professional	Sex	Role	Resource
Education 1	M	Headteacher and teacher	Special school for 3- to 21-year-olds
Education 2	M	Psychologist	Special school for 16- to 21-year-olds
Education 3	F	Headteacher	Special school for 3- to 21-year-olds
Education 4	F	Headteacher	Special school for 3- to 21-year-olds
Education 5	F	Headteacher	Special school for 12- to 21-year-olds
Education 6	F	Teacher and academic tutor	Inclusive resource at non-special school Age range: 12–16 years old
Education 7	F	Teacher and academic tutor	Inclusive resource at non-special school Age range: 13–16 years old
Education 8	M	Teacher	Hospital classroom for 11- to 18-year-olds
Healthcare 1	F	Midwife	Primary and hospital care
Healthcare 2	M	Psychiatrist ^1^	Outpatient clinic
Healthcare 3	F	Social worker ^1^	Inpatient care
Healthcare 4	M	Clinical Psychologist ^1^	Inpatient care
Healthcare 5	F	Social worker ^1^	Outpatient clinic
Healthcare 6	F	Psychiatrist ^1^	Outpatient clinic
Healthcare 7	F	General adult psychiatrist	Outpatient clinic and inpatient care
Healthcare 8	F	Psychiatrist ^1^	Outpatient clinic
Healthcare 9	F	Nurse ^1^	Outpatient clinic
Healthcare 10	F	Clinical psychologist ^1^	Outpatient clinic and inpatient care

M: male; F: female; ^1^ healthcare for 5- to 21-year-olds.

**Table 2:** Dimensions and associated questions.

Dimensions	Questions
Family	What questions does the family ask with regards to situations relating to affectivity and sexuality? What problems do care providers encounter when managing the socio-affective needs of young people with ASD? What role does the family play in sex and affective education, and what implications does it have?
Training	Is there specific professionals training on affectivity and sexuality? Is there refresher training on affective and sexual behaviours when working with individuals with ASD?
Sex education	Are there programmes or protocols in place to facilitate sex and affective education among these young people? What support systems do you have in place at your centre to facilitate this type of health education?
Sexuality of individuals with ASD	Do you think that young people with ASD behave differently on an affective and/or sexual level compared to other adolescent groups?

**Table 3:** Themes and categories identified from education professionals.

Theme	Category
Family coping	-Family beliefs -Family difficulties -Family denial -Fear -Role of siblings
Training for education professionals	-Training received -Identifying educational needs
Curricular and collaborative strategies	-Cross-sectional and curriculum-integrated area -Coordination with families -Multidisciplinary collaboration with healthcare staff
Profile of adolescents with ASD with regards to their affective/sexual development	-Inappropriate social behaviour -Risky behaviour -Communication problems -Problematic and dysfunctional sexuality -Acquiring knowledge

**Table 4:** Themes and categories identified from healthcare professionals.

Theme	Category
Experiences with the family	-Types of consultations -Cultural diversity
Specialised training of healthcare professionals	-Training -Sexual/affective exploration during consultations
Profile of adolescents with ASD	-Lack of knowledge among individuals -Interpretation of relationships -Vulnerability

## Data Availability

Please contact the corresponding author (judith.roca@udl.cat, carme.campoy@udl.cat) to request data.
